# Tree diversity and its ecological importance value in organic and conventional cocoa agroforests in Ghana

**DOI:** 10.1371/journal.pone.0210557

**Published:** 2019-01-11

**Authors:** Michael Asigbaase, Sofie Sjogersten, Barry H. Lomax, Evans Dawoe

**Affiliations:** 1 Department of Agriculture and Environmental Science, School of Biosciences, University of Nottingham, Nottingham, United Kingdom; 2 Department of Agroforestry, Faculty of Renewable Natural Resources, Kwame Nkrumah University of Science and Technology, Kumasi, Ghana; Leiden University, NETHERLANDS

## Abstract

Cocoa agroforestry systems have the potential to conserve biodiversity and provide environmental or ecological benefits at various nested scales ranging from the plot to ecoregion. While integrating organic practices into cocoa agroforestry may further enhance these potentials, empirical and robust data to support this claim is lacking, and mechanisms for biodiversity conservation and the provision of environmental and ecological benefits are poorly understood. A field study was conducted in the Eastern Region of Ghana to evaluate the potential of organic cocoa agroforests to conserve native floristic diversity in comparison with conventional cocoa agroforests. Shade tree species richness, Shannon, Simpson’s reciprocal and Margalef diversity indices were estimated from 84 organic and conventional cocoa agroforestry plots. Species importance value index, a measure of how dominant a species is in a given ecosystem, and conservation status were used to evaluate the conservation potential of shade trees on studied cocoa farms. Organic farms recorded higher mean shade tree species richness (5.10 ± 0.38) compared to conventional farms (3.48 ± 0.39). Similarly, mean Shannon diversity index, Simpson’s reciprocal diversity index and Margalef diversity index were significantly higher on organic farms compared to conventional farms. According to the importance value index, fruit and native shade tree species were the most important on both organic and conventional farms for all the cocoa age groups but more so on organic farms. Organic farms maintained 14 native tree species facing a conservation issue compared to 10 on conventional cocoa farms. The results suggest that diversified organic cocoa farms can serve as reservoirs of native tree species, including those currently facing conservation concerns thereby providing support and contributing to the conservation of tree species in the landscape.

## Introduction

Cocoa agroforestry is a production system in which farmers intentionally integrate shade trees with cocoa trees on the same plot together with food crops. Since cocoa agroforests are in many ways–e.g. in terms of tree cover, composition and structure–closer to natural forest ecosystems compared to monocultures it is receiving a lot of attention following the realisation of its potential to conserve biodiversity and provide environmental, biological, ecological and socio-economic benefits at various nested scales such as plot, farm, landscape and ecoregion [1–4]. Cocoa agroforests conserve native plant and animal diversity [5] and provide co-products which diversify farmers’ diets as well as supplementary income and some security from climate change related shocks [6–8]. As future climate predictions suggest a reduction of suitable cocoa production areas in both Ghana and Côte d’Ivoire [9] the two major cocoa producers, cocoa agroforests may play a significant role in sustaining cocoa production in these countries as these systems have the potential to improve micro-climatic conditions thus enhancing their ecological resilience [6; 10].

There is a growing global demand for cocoa and it is estimated that in the next decade world cocoa production and price will rise by 10% and 25% respectively [11]. To meet this demand, farmers’ immediate response, as it had been in the past, includes intensification and/or expansion of cocoa production systems, both of which have been cited as drivers of deforestation and declining biodiversity in West Africa and elsewhere [4; 6; 10]. Cocoa is mostly grown under partially cleared forests; the retained trees provide shade for cocoa and co-products for farmers while shade tree leaf litter inputs and accumulated nutrients in forests soil ensure productivity [10; 12]. However, farmers gradually replace native trees with food crops or fruits (e.g. *Citrus spp*. and *Musa spp*.) and plant more cocoa as the cocoa trees mature to increase income [9; 13]. This trend of simplification within cocoa agroforests leads to the creation of agrochemicals-dependent cocoa systems referred to as conventional cocoa systems, which smallholders cannot manage due to high input costs [12; 14]. Therefore, cocoa intensification within the current socio-economic context of cocoa farmers may result in shifts in cocoa production to new frontiers through clearance of forests land thus enhancing deforestation. Moreover, use of synthetic agrochemicals in the conventional cocoa systems may negatively modify soil biota composition which could affect soil health that underpins productivity [15].

Organic farming defined as production systems with an inherent ethos to sustain the health of soils, ecosystems and people prohibits the use of synthetic agrochemicals [8; 15]. Since synthetic agrochemicals such as fertilizer, pesticides and herbicides are not used in organic farming, farmers pursue one of two strategies; organic monocultures or organic agroforests. Organic cocoa monocultures rely solely on organic agrochemicals for soil nutrient replenishment and control of weeds, pests and diseases. Organic cocoa agroforests make use of a variety of shade trees to suppress weed growth and insect pest outbreaks [16; 17] and to compensate for nutrient losses due to nutrient uptake by cocoa trees through nitrogen fixation, reduced nutrient leakage and decomposition of litter from shade trees [17; 18]. Cocoa trees also benefit from microclimate amelioration and increased water retention [4; 6; 16]. Additionally, the shade trees provide a range of benefits to people, soils and ecosystems such as; (i) provision of food and fruits [7], (ii) enhanced soil and water quality through reduced erosion and pollution [16], (iii) maintenance of high levels of native species and functional agrobiodiversity [7], (iv) increased farm resilience [8] and carbon sequestration [4; 5; 6; 7] and its attendant climate change mitigation benefits.

Therefore, organic cocoa farming that makes use of diverse shade trees (agroforestry) might contribute meaningfully to the mitigation of biological diversity loss in the tropics [8; 19], especially in regions where forest cover has been significantly reduced [1]. Furthermore, it has been shown that an integration of organic farming (i.e. the use of solely organic inputs) and agroforestry (introduction of trees on farmlands) would enhance biodiversity conservation [2; 8; 15] and integration has been strongly recommended for Africa [2; 20]. However, robust data supporting this claim are lacking, especially in tropical and developing countries [20; 21]. In Ghana, the integration of organic farming practices into shade cocoa production (i.e. organic cocoa agroforestry) is not a recent phenomenon as systems date back to 1870 when cocoa was first introduced but organic certification is a relatively new phenomenon. While conventional cocoa production has been carried out using shade systems, and in some cases without shade trees (full sun), fertilisation of cocoa plots and the control of pests and diseases have been undertaken using inorganic inputs. The recruitment of shade tree species in cocoa systems is a reflection of what farmers deem important for the provision of shade and other ecological services [13; 16]. Thus, it is essential to understand the importance value–a measure of how dominant a species is in a given ecosystem–of shade tree species in cocoa agroforestry systems. The composition and structure of cocoa agroforestry systems changes with time via management, thus dominant shade tree species at each stage as the cocoa systems mature is closely linked to management [13; 22].

There is a consensus that agroforestry systems can conserve flora and fauna better than monocultural crop systems, but not native forests (e.g. [4; 5; 6; 10; 16]). However, little is known about organic cocoa agroforests contribution to the conservation of flora and fauna, making it difficult to quantitatively evaluate their contribution to the conservation of floristic diversity but such data is vital for biodiversity conservation because the major cocoa production areas are also classified as biodiversity hotspots [1; 2; 16; 20]. Moreover, given the large spatial extent of cocoa systems in the major cocoa production countries [1; 9], the significant overlap with biodiversity hotspots [1; 2; 16] and the debate on land-sparing or land-sharing species conservation strategies [9; 23], it is crucial to evaluate the potential of cocoa systems to conserve tree diversity. It has been observed that the adoption of land-sparing and agroecological methods like cocoa agroforestry can create a more biodiversity-friendly agricultural matrix [16; 24] to develop complex, multilayered habitats, and improve connectivity thus exhibiting potential for a providing a solution to the biodiversity-food trade-off [25].

There is also a growing consumer demand for organic commodities coupled with advocacy by environmentalists for organic cocoa because such systems tend to be more ecologically sustainable compared with conventional systems [7; 8; 19]. Yet, in West Africa the comparison of shade organic cocoa systems and conventional cocoa systems in terms of biodiversity benefits is not well documented. The present research seeks to bridge these gaps and to contribute to the understanding of the benefits of organic cocoa agroforests in terms of conservation of native shade tree species. Specifically, the study compared the community structure (abundance, heterogeneity, richness and composition) of organic and conventional farms. It also determined the conservation status and importance value of shade species (i.e. both woody and non-woody shade providing plants) in both production systems. Finally, it determined the shade strategies (the stem density/number and type of shade species planted/retained on cocoa farms) utilized by farmers. We hypothesized that organic cocoa farms will be more diversified in structure and richer in shade species composition compared to conventional farms.

## Methodology

### Study area

**Fig 1:**
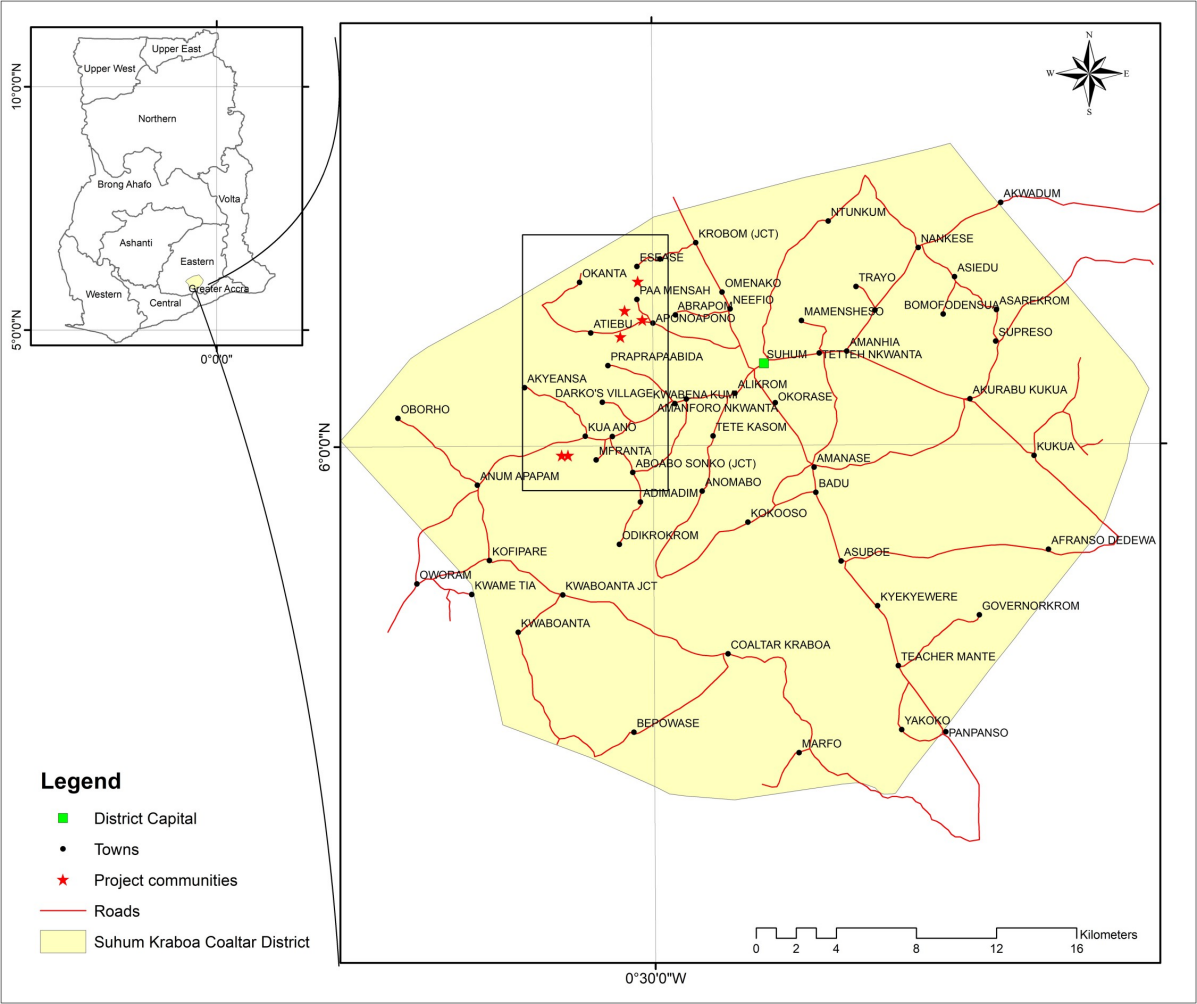
Map of the study area.

The study was conducted in seven randomly selected cocoa growing communities (Nsuta, Owawase, Safrosua, Sebiase, Yaw Kwapong, Abeho and Kuano) in the Suhum Municipality in the Eastern Region of Ghana (Fig 1). Suhum lies about 60 km north-north-west of Accra (the capital of Ghana) at N 6^o^ 5′ and W 0^o^ 27′ and is about 400 km^2^ [26; 27]. Cocoa farming in Ghana originated from the Eastern Region [13; 22], the part of Ghana where Suhum is located and Suhum harbours the country’s oldest organic cocoa farms as it was pioneered in this area.

Ecologically, Suhum lies within the semi-deciduous forest zone but anthropogenic activities such as agriculture, logging and extraction of fuelwood have reduced the original vegetation to an insignificant level and the land is now mainly covered by fallows and secondary forests [23]. The shaded-cocoa systems in the area are mostly mixed stands of cocoa with variable proportions of naturally generated upper canopy shade trees such as *Terminalia superba* Engl. & Diels, *Entandophragma angolense* (Welw.) C.DC, *Alstonia boonei* de Wild, *Antiaris toxicaria* Lesch.and *Spathodea campanulata* P. Beauv. Increasingly, fruit trees including orange (*Citrus sinensis)* (L.) Osbeck, Avocado (*Persea americana* Mill.) and mango (*Mangifera indica* L.) are planted for shade, food and other purposes. The cocoa farms are generally small-scale in nature typically not exceeding 2 hectares. Cocoa trees are planted at a spacing of 3 m × 3 m giving a planting density of approximately 1100 trees/hectare with majority of farms having the recommended 12–18 shade trees *per* hectare corresponding to 30–40% canopy cover [22]. The major management practices undertaken are shade control, fertiliser application, weeding, pest and disease control, harvesting of pods and processing of beans. Currently, the Cocoa Research Institute of Ghana recommends the application of specially formulated cocoa fertiliser “Asaase wura” (0-22-18+ 9Ca+7S+6MgO) [28] at the rate of 7.5 bags (375 kg ha^-1^) and “Nitrobor” (Nitrogen + boron) at the rate of (2.5 bags) 125 kg ha^-1^. The cocoa farms in the area are either organically or conventionally managed. Organic farming prohibits the use of synthetic agrochemicals such as inorganic fertilizers, pesticides, herbicides and fungicides and encourages the use of manure, mulch, organic fertilizer and organic pesticides while conventional farming uses synthetic agrochemicals. Both organic and conventional cocoa farmers in the study area maintain shade trees on their cocoa farms. Yields from both systems vary over a wide range ranging from 400–800 kg ha^-1^ (*Personal com* and [23]). In this study we define organic cocoa farms as cocoa agroforests (shade cocoa) that have been managed for at least five years using only certified organic inputs (i.e. organic fertilizer, organic pesticides and herbicides) to produce cocoa beans that meet international standards for the production of organic cocoa. All the organic cocoa farmers were registered and certified by Control Union, an international certification body active in more than 70 countries.

Rainfall in the study area ranges from 1270 mm to 1651 mm and is bimodal, i.e. major season (April to July) and minor season (September to November) with a dry spell or main dry season (November to March). Average temperature ranges from 24^o^ C to 29^o^ C, relative humidity for the rainy and dry seasons ranges from 87% to 91% and 48% to 52% respectively [23]. The municipality is rain fed agrarian, with cocoa farming being the major occupation; other sources of livelihood include cultivation of food crops, poultry, forestry and trading. Soils in the study area were formed from similar thoroughly weathered parent material; they are porous, well drained and loamy and are grouped under forest ochrosols [23; 29]. The ecology of the study area is similar to what pertains in the major cocoa growing areas in Ghana [30; 31] and is therefore broadly representative.

### Selection of cocoa farms

We adopted a multi-stage approach in the selection of study communities and farms/farmers for the study. First the Suhum Municipality was purposively selected because the production of organic cocoa beans in Ghana was pioneered here and the oldest organic cocoa farms can be found within the Municipality. Next seven cocoa producing communities within the Municipality known to have farmers producing organic cocoa were randomly selected from a list obtained from local offices of the Ghana COCOBOD (regulators of the cocoa sector). Organic and conventional cocoa farmers/farms were then randomly selected from separate lists provided by the regulators. Where a farmer had more than one of a particular farm type, only one was selected randomly. Conventional farms are cocoa agroforest managed using inorganic inputs. Both the organic and conventional cocoa farms were categorised into three cocoa age groups namely; Young Cocoa Systems (YCS, ≤ 15 years), Mature Cocoa Systems (MCS, 16 to 30 years) and Old Cocoa Systems (OCS, ≥ 31 years) and 14 cocoa farms were selected *per* cocoa age group *per* farm type (i.e. overall, 42 organic and 42 conventional farms were selected). Land preparation methods, management practices and cropping history of farms were similar. All the selected sites were neighbouring communities except two which were located 8–10 km away. All selected farmers/landowners agreed to participate in the research and no further permission was required.

### Species inventory and quantitative measurement

The area of the farm was obtained by walking along its perimeter with a Global Positioning System (Garmin GPSMAP 62s) after which a 25 m x 25 m plot was then randomly established [13; 30]. Shade trees and shrubs were identified to the species level (botanical and local names) with the help of an experienced forest technician (from the Council for Scientific and Industrial Research) and two local informants and after Hawthorne and Jongkind [31]. The uses of all the shade species were also determined and recorded. The circumference of the stem of all shade species and cocoa trees (>5 cm) were measured at 1.3 meters above the ground with a tape measure in centimetres and later converted to diameter values. For multi-stemmed plants, each stem was measured and the equivalent diameter of the plant estimated by taking the square root of the sum of the diameter squared of all stems *per* plant [32]. All data were collected between April and August 2016.

### Data processing and estimation of quantitative parameters

#### Shade trees species diversity and richness

Shade trees species richness (hereafter species richness) *per* plot was estimated by counting the number of species in each plot (i.e. observed species richness). The total species richness on organic and conventional farms in each cocoa age-group was calculated using two non-parametric estimators; (i) second-order Jacknife [33], which is based on the observed frequency of rare species and minimises the bias of using observed species richness as an estimator and (ii) the singletons and doubletons of Chao [34], which provides a lower bound estimate of species richness. The Chao1 total richness (S_chao1_) and the second-order Jackknife (S_jk2_) were calculated as S_chao1_ = S_o_ + [a^2^/(2b)] and S_jk2_ = S_o_ + 2a –b, where S_o_ is observed species richness, a is the number of singletons and b is the number of doubletons (b > 0).

Shannon diversity index (H′), Simpson’s reciprocal index (1/D) and Margalef’s diversity index (Dmg) [35] were estimated in all sampled plots and used together to provide an assessment of the richness and diversity of the shade trees in the two systems. Shannon diversity index is weighted towards rare species, independent of sample size commonly used in biodiversity surveys and combines both species abundance and richness thus comparison with other studies can be done with ease [35; 36]. The Shannon diversity was calculated as; H′ = -∑pi*(Ln *pi*), *pi* is equal to n_i_/N, where n_i_ is the number of individuals *per* species *i* and N is the total number of individuals *per* study plot thus *pi* is the proportion of individuals in species *i*. The Simpson’s reciprocal index gives more importance to species abundance and takes into account both species richness and evenness; it was estimated using the formula 1/D = 1/Σ[(n(n-1))/N(N-1)], where n is the total count of individuals for a particular species in the sample and N is the total count of individuals in the sample. The Margalef index, which is weighted towards species richness and has no limit value but is sensitive to sample size, was calculated as D_mg_ = (S-1)/In N, where S is species richness and N is the number of individuals.

#### Spatial structure and composition of cocoa farms

Species composition of the two cocoa production systems in each cocoa age group was assessed *via* Jaccard and Sørensen indices, both of which weight matches and mismatches using species presence/absence data sets [35]. To compare spatial structure, all shade providing species were grouped into trees (trees not maintained for food or fruits e.g. *Milicia excelsa*) and food and fruits (trees/crops maintained for fruits or food e.g. *Citrus spp*. and *Musa spp*). Stem densities and basal areas of all shade providing species–as well as that of cocoa were estimated. In order to assess how shade trees/species were managed by the farmers, all associated trees were grouped into domestic (trees maintained to meet domestic needs e.g. food, medicine, etc), ecological (trees maintained to provide shade, fix nitrogen, etc) and economic (trees maintained for income e.g. timber *spp*.) based on their uses.

#### Shade tree species conservation status and ecological importance

Shade tree species with conservation interest were checked using the IUCN Red List of Threatened Species [37] and in-country star categories for species with conservation priority [38]. The IUCN Red List categories include; critically endangered (CR), endangered (EN), vulnerable (VU) and near threatened (NR), repectively defined as species whose risk of extinction in the wild was imminent, extremely high, high and likely in the future. The in-country star categories include; (i) Black star–species require urgent conservation attention because it is globally rare and nationally uncommon, (ii) Gold star–species needs conservation attention because it is fairly rare worldwide and/or nationally, (iii) Blue star–species needs protection because it is rare nationally and common globally or vice-versa, (iv) Scarlet star–species requires urgent control measures because though the species is nationally common it is facing high exploitation pressure and (v) Red star–species needs some control measures because though the species is nationally common it is facing exploitation pressure [38].

The Importance Value Index (IVI) of each species was estimated as IVI = RA+RD+ RF, where RA is relative abundance calculated as the number of individuals *per* species *per* hectare, RD is relative dominance defined as the basal area *per* species *per* hectare and RF is relative frequency (*per* ha) estimated as the proportion of plots in a cocoa production system where the species occurred at least once. The IVI which was developed by [39] was used in this study as a proxy for ecological importance of shade species and the composition of dominant species in organic and conventional cocoa systems were assessed by comparing ten species with high IVI [3].

### Statistical analysis

Data conforming to the assumptions of ANOVA were assessed using residual plots and two-way analysis of variance (ANOVA) was used to assess statistical differences between farm types (Org. vs. Con.) and cocoa age-groups (Young, Mature and Old); least significant difference (LSD) post hoc test was conducted where there was significant difference among cocoa age-groups. A Kruskal-Wallis test (K-W ANOVA) was used where the data were not normally distributed. A Chi-Square test was used to check if there was an association between cocoa age-groups or farm type and tree use group in terms of species richness and stem density. All the data were processed and analyzed using GenSat (version 17.1). Differences between assessed indices and variables in the two cocoa production systems were considered significant at *p* < 0.05.

## Results

### Shade tree species abundance and importance value

In the YCS, 454 individuals belonging to 41 species and 18 families were found in the organic systems whereas 198 individuals belonging to 36 species and 18 families were recorded in the conventional systems. The organic farms of the MCS recorded 387 individuals, 41 species and 22 families whereas 182 individuals, 35 species and 17 families were documented on the conventional MCS farms. The organic OCS recorded 19 families, 38 species and 299 individuals whereas the conventional OCS had 17 families, 27 species and 114 individuals. All recorded shade trees were native species, except *Cedrela odorata* L., *Artocarpus altilis* (Parkinson) Fosberg, *Gliricidia sepium* (Jacq.) Walp. and *Leucaena leucocephala* (Lam.) de Wit.

The food and fruit species *Musa sapientum* L. f. thomsonii King ex Baker, *Musa paradisiaca* L., *Mangifera indica*, *Carica papaya* L., *Citrus sinensis* and *Persea americana* were present in all the studied organic farms. Additionally, *Chrysophyllum subnudum* (Bak.) which is an important forest fruit for domestic consumption with potential for local and international markets were found on the mature and old organic farms. *Carica papaya*, *Musa sapientum*, *Musa paradisiaca* and *Persea americana* occurred on all the studied conventional farms. *Citrus aurantifolia* (Christm.) Swingle, *Citrus sinensis*, *Cocos nucifera* L., *Mangifera indica* and *Psidium guajava* (L.) were found in at least one of the three cocoa age groups. The relative abundance of food and fruit species (*per* ha) ranged from 77.5% to 79.8% on organic farms and 45.4% to 63.8% on conventional farms (S1 Table).

Important timber species such as *Terminalia ivorensis* (A. Chev.) and *Entandrophragma angolense* (Welw.) C.DC. and *Ficus sur* Forssk., a lesser utilized timber species, were also relatively dominant in the organic MCS, whereas *Alstonia boonei* and *Morinda lucida* Benth., both important medicinal plants in West Africa, were relatively dominant in the organic OCS. Forest trees such as *Holarrhena floribunda* (G. Don) Dur and Schinz and *Morinda lucida* were also relatively abundant on the young conventional farms. The relative abundance of nitrogen fixing trees was 6% ha^-1^ on organic farms whilst that of conventional farms was 2.6% ha^-1^.

According to the IVI, the ten most abundant shade species represented 71.2%, 71.6% and 70.8% of recorded species in the organic YCS, MCS and OCS respectively and 67.1%, 70.0% and 68.3% of the species found in the conventional YCS, MCS and OCS respectively (S2 Table). The most important species on the organic farms (i.e. YCS, MCS and OCS) were the food and fruit species *Musa spp*., *Citrus sinensis*, *Persea americana* and *Magnifera indica*, valuable timber species *Terminalia ivorensis*, *Milicia regia* (A.Chev.) Berg and *Entandrophragma angolense*, and important medicinal species *Alstonia boonei*, *Morinda lucida* and *Holarrhena floribunda*. Other ecologically and economically important species noted among the ten most important species were *Newbouldia laevis* (P. Beauv.) Seemann ex Bureau, *Spathodea campanulata*, *Sterculia tragacantha* Lindl., *Ficus exasperata* Vahl and *Ficus sur*. For the conventional farms (i.e. YCS, MCS and OCS), the food and fruit species *Musa spp*., *Citrus spp*., *Carica papaya*, *Cocos nucifera* and *Persea americana* were found to be the most abundant. Some valuable timber species such as *Milicia excelsa* (Welw.) C.C.Berg, *Antiaris toxicaria* and *Terminalia ivorensis* were among the ten most important species (S2 Table). Medicinal plants such as *Voacanga africana* Stapf, *Morinda lucida*, *Holarrhena floribunda* and forest species *Rauvolfia vomitoria* Afzel, *Lonchocarpus sericeus* (Poir.) Kunth ex DC. and *Ficus exasperata* were notably important on the conventional farms.

### Shade tree species richness and diversity

**Fig 2:**
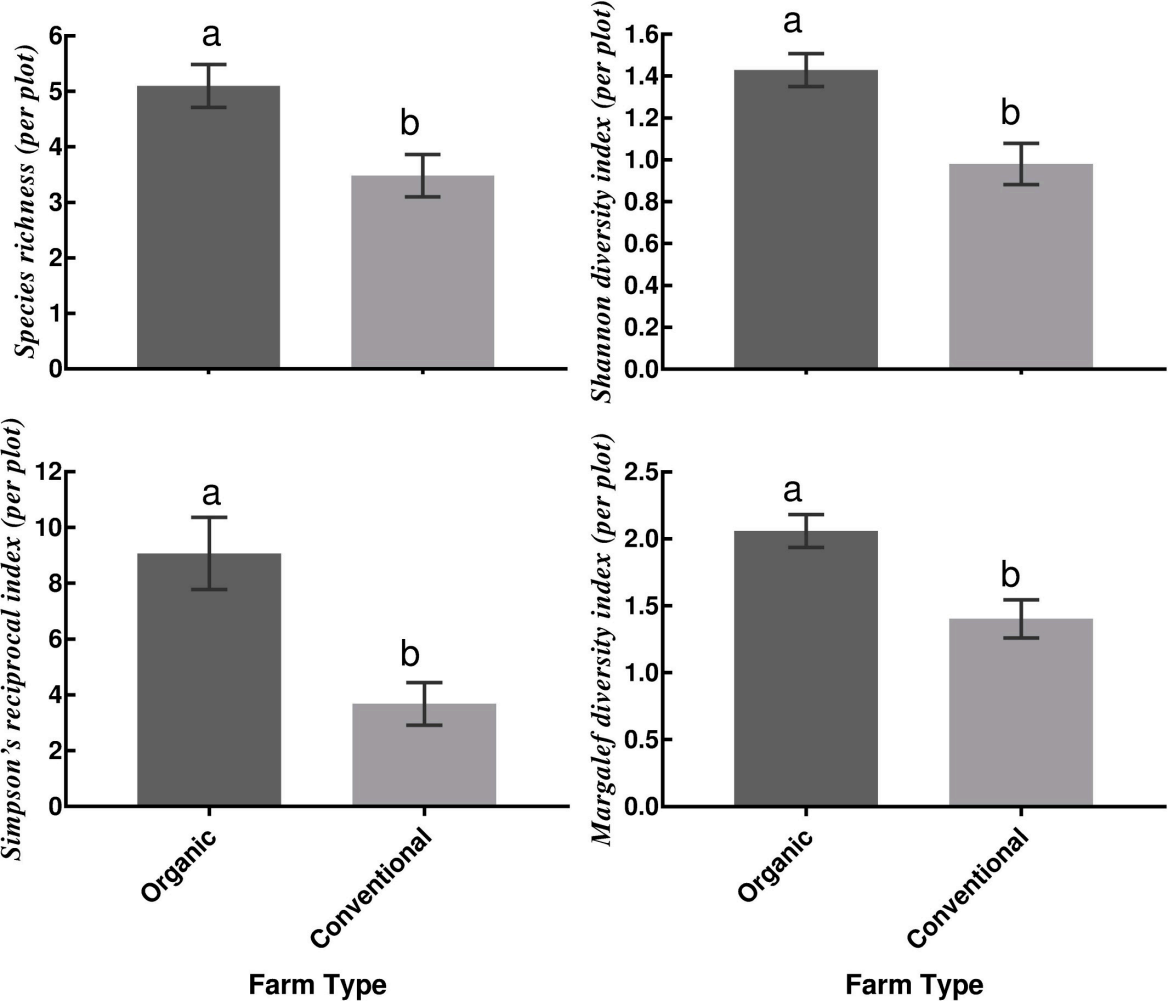
Mean shade species richness and diversity indices (*per* plot ± SEM, n = 42) between studied organic and conventional cocoa systems. Bars of Farm Type (Organic vs. Conventional) with different letters implies significant differences at α = 0.05.

The number of shade tree species *per* plot on the organic farms ranged from 2–13 with an average of 5.10 ± 0.38 and on the conventional farms, a range of 0–13 species with an average of 3.48 ± 0.39 was recorded. The total estimated species richness based on Chao1 and the second-order Jackknife were 111.68 ± 21.53 and 119 respectively for the organic farms and 90.80 ± 17.99 and 99 for the conventional farms in the studied cocoa systems. Organic farms had significantly higher mean species richness (F_1, 78_ = 8.91, *p* = 0.004), Shannon diversity index (F_1, 78_ = 12.80, *p* < 0.001), Simpson’s reciprocal diversity index (F_1, 78_ = 12.25, *p* < 0.001) and Margalef diversity index (F_1, 78_ = 12.22, *p* < 0.001) when compared to conventional farms (Fig 2). Average species richness and diversity indices values were similar across the different cocoa-age groups.

The richest families in the organic YCS were Moraceae (8 *spp*.), Sterculiaceae (5 *spp*.), Fabaceae (4 *spp*.) and Apocynaceae (4 *spp*.) while Moraceae (7 *spp*.), Fabaceae (4 *spp*.) and Apocynaceae (4 *spp*.) were the richest families for the conventional YCS. Few species (1–3) were observed on all the other families found on organic and conventional farms. For the MCS, Moraceae and Fabaceae (6 *spp*. each) were the richest families on both the organic and conventional farms; the other documented families recorded ≤ 3 *spp*. *per* family. However, in the organic OCS, three families Fabaceae (6 *spp*.), Moraceae (5 *spp*.) and Apocynaceae (4 *spp*.) were the richest. In conventional OCS, the richest documented families were Moraceae and Apocynaceae (4 *spp*. each).

### Spatial structure and composition of organic and conventional cocoa farms

On organic farms there was a threefold increase in the density of food and fruits shade trees (*per* ha) when compared to conventional farms (Org. 341 ± 38 vs. Con. 106 ± 18) and the mean-ranks of fruit trees density were significantly different between the farm types (H = 29.88, df = 1, *p* < 0.001). Both shade tree density and total density did not differ significantly between organic and conventional farms but the density of cocoa trees (Org. 1012 ± 40 stems ha^-1^ vs. Con. 1203 ± 40 stems ha^-1^) were significantly different (F_1, 78_ = 11.67, *p =* 0.001). The density of shade trees which were timber species was significantly higher on organic farms compared to conventional farms (Org. 68 ± 7 vs. Con 40 ± 7; H = 11.05, df = 1, *p* < 0.001) but that of non-timber species was similar for both farm types.

**Fig 3:**
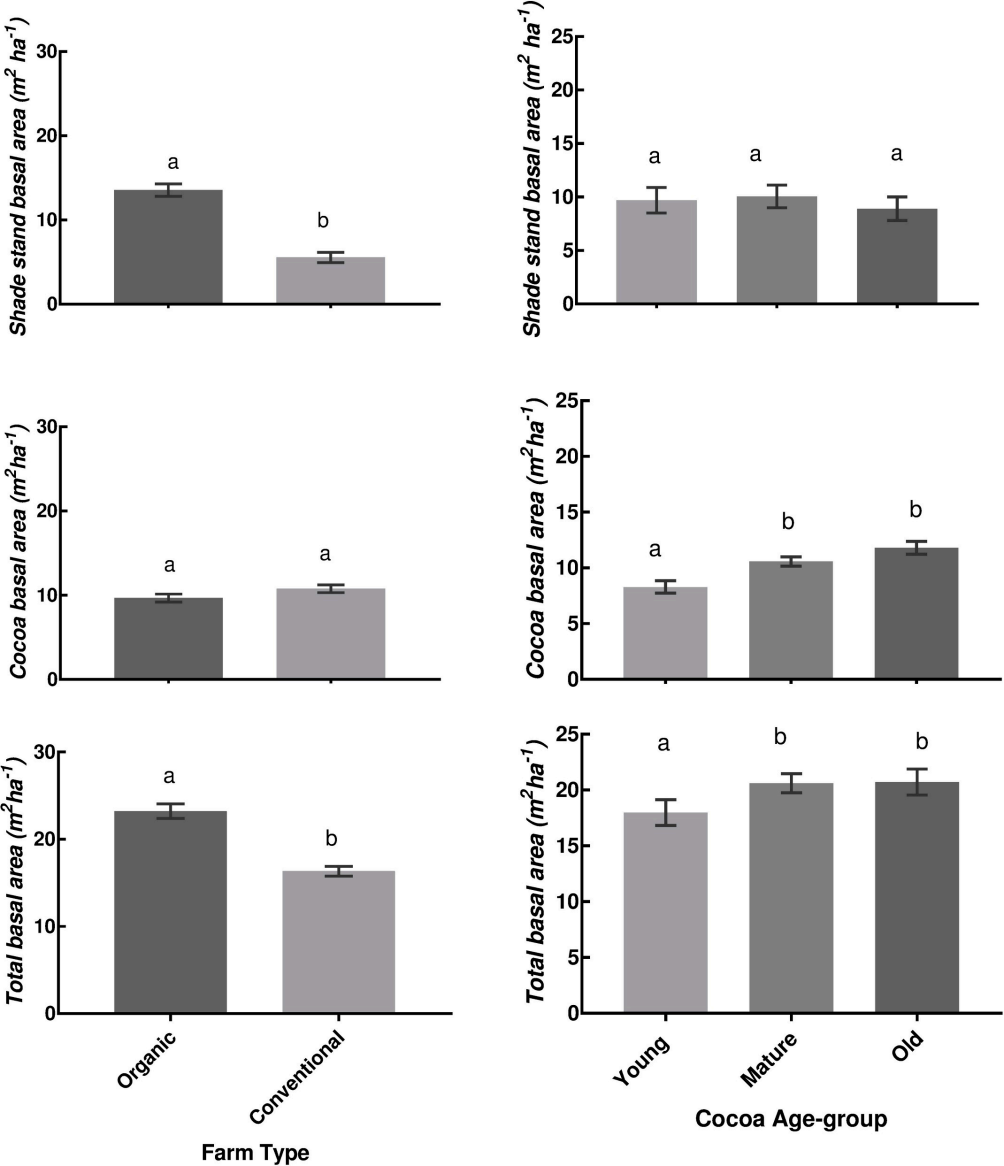
Shade stand, cocoa and total basal areas (mean ± SEM) for farm type (n = 42) and cocoa age-groups (young, mature and old, n = 28). Bars of Farm Type or Cocoa Age-group with different letters implies significant differences at α = 0.05.

In terms of the basal area, the mean shade trees and total basal areas were significantly higher on organic farms than conventional farms (Fig 3; Shade trees; F_1, 78_ = 70.80, *p* < 0.001, Total basal area; F_1, 26_ = 49.05, *p* < 0.001) but the mean cocoa basal areas were similar. Across the different cocoa age-groups, there was a significant difference between the mean values for cocoa basal area (F_2, 78_ = 11.93, *p* < 0.001) as well as mean total basal area (F_2, 78_ = 3.32, *p =* 0.041); Least Significant Difference (LSD) post hoc test showed that both the mature and old cocoa systems had higher mean cocoa basal area and total basal area compared to the young cocoa farms. Similar mean shade trees basal area was recorded across the cocoa age-groups. Overall, organic farms were larger than conventional farms, 1.71 ± 0.26 and 1.02 ± 0.19 ha, respectively; the Kruskal Wallis mean-ranks of farm size values differed significantly (H = 8.663, df = 1, *p* = 0.003) between organic and conventional types.

**Fig 4:**
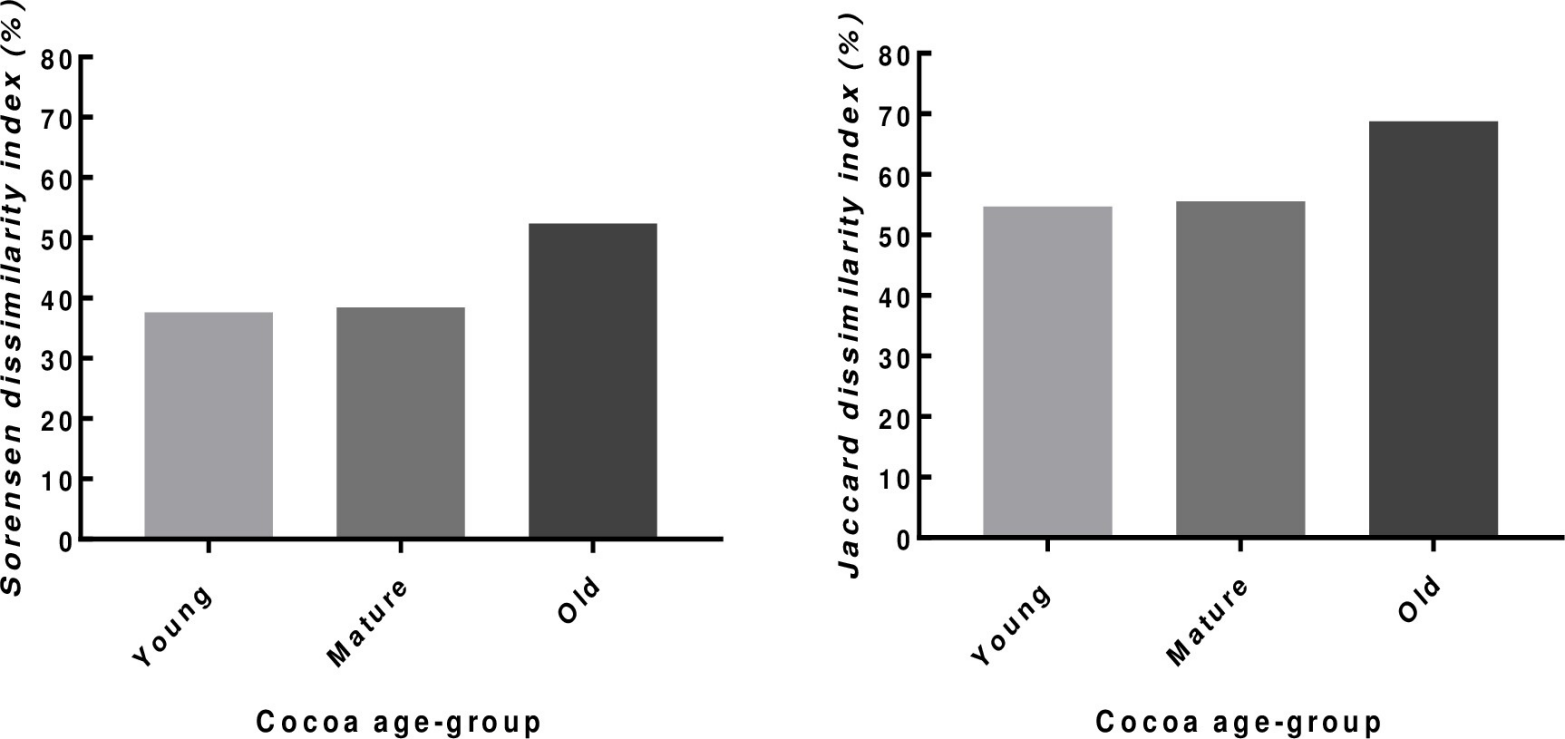
Mean species dissimilarity between organic and conventional farms (n = 14) in each cocoa age-group (young, mature and old).

Species dissimilarity between organic and conventional systems was greatest for both Jaccard and Sorrosen dissimilarity indices in the OCS and least in the YCS (Fig 4).

### Shade tree species conservation status and ecological importance

Overall, in the organic cocoa systems, a total of 68 species were identified out of which eight species were either endangered, vulnerable or near threatened (Table 1). Three of these eight species *Albizia ferruginea*, *Entandrophragma angolense* and *Pterygota macrocarpa* were only found on organic farms. Three vulnerable species, *Milicia regia Entandrophragma angolense* and *Terminalia ivorensis* were among the ten most ecologically important species on the organic farms. Seven species out of the 57 species recorded in the conventional systems were either endangered, vulnerable or near threatened; two of these seven species *Cedrela odorata* and *Antrocaryon micraster* occurred on only the conventional farms (Table 1).

**Table 1:** List of shade species with conservation concern and their relative abundance (R.A), relative dominance (R.D), relative frequency (R.F) and importance value indices (IVIs). The list was generated based on IUCN Red List of Threatened Species and in-country star categories for species with conservation priority.

Tree species	R.A (%)	R.D (%)	R.F (%)	IVIs	Conservation status	
					In-Country	IUCN
**Organic cocoa farms**						
*Albizia ferruginea* (Guill. and Perr.) Benth.^*a*^	0.09	0.13	0.37	0.59	Scarlet	Vulnerable
*Albizia glaberrima* (Schumach. and Thonn.) Benth.	0.22	0.77	1.04	2.03	Green	Least concern
*Ehretia trachyphylla* C.H.Wright	0.09	0.04	0.37	0.50	Gold	
*Entandrophragma angolense* (Welw.) C.DC.^*a*^	1.40	1.00	4.46	6.87	Red	Vulnerable
*Erythrina vogelii* Hook f.	0.35	0.25	0.74	1.34	Blue	
*Mansonia altissima* (A.Chev.) A.Chev.	0.52	0.22	1.09	1.83	Pink	Endangered
*Milicia excels*a (Welw.) C.C.Berg	0.09	0.01	0.37	0.47	Scarlet	Near threatened
*Milicia regia* (Welw.) C.C.Berg	0.96	1.47	2.60	5.04	Scarlet	Vulnerable
*Nesogordonia papaverifera* (Chev, A.) Cap.	0.35	0.18	1.12	1.65	Pink	Vulnerable
*Pterygota macrocarpa* K.Schum.^*a*^	0.09	0.01	0.37	0.47	Red	Vulnerable
*Ricinodendron heudelotii* (Baill.) Pierre ex Heckel^*a*^	0.26	0.70	1.12	2.08	Scarlet	
*Synsepalum dulcificum* (Schum. and Thonn.) Daniell^*a*^	0.09	0.00	0.37	0.46	Blue	
*Terminalia ivorensis* A.Chev.	1.67	1.89	3.72	7.28	Scarlet	Vulnerable
*Triplochiton scleroxylon* K.Schum.^*a*^	0.09	0.20	0.37	0.66	Scarlet	
**Conventional cocoa farms**						
*Albizia glaberrima* (Schumach. and Thonn.) Benth	0.20	0.24	0.55	0.99	Green	Least concern
*Antrocaryon micraster* A.Chev.^*b*^	0.20	0.12	0.55	0.88	Red	Vulnerable
*Cedrela odorata* L.^*b*^	0.20	0.02	0.55	0.77	Others	Vulnerable
*Ehretia trachyphylla* C.H.Wright	0.82	0.24	1.64	2.70	Gold	
*Erythrina vogelii* Hook f.	0.20	0.12	0.55	0.87	Blue	
*Mansonia altissima* A.Chev.) A.Chev.	0.20	0.08	0.55	0.83	Pink	Endangered
*Milicia excelsa* (Welw.) C.C.Berg	1.02	0.63	2.19	3.84	Scarlet	Near threatened
*Milicia regia* (Welw.) C.C.Berg	0.61	0.08	1.09	1.78	Scarlet	Vulnerable
*Millettia zechiana* Harms^*b*^	2.66	0.35	2.73	5.74	Green	Least concern
*Nesogordonia papaverifera* (Chev, A.) Cap.	0.41	1.17	1.09	2.67	Pink	Vulnerable
*Terminalia ivorensis* A.Chev.	0.82	0.59	2.19	3.60	Scarlet	Vulnerable

a = species found on only organic farms

b = species found on only conventional farms

According to the star categories of species with conservation priority, organic farms recorded a relatively higher number of species for the Blue, Scarlet and Red categories than the conventional farms. Six of the species with in-country species conservation priority occurred on only organic farms, one on only conventional farms and seven on both farm types. Overall, 14 and 10 native tree species with conservation concern were recorded on the organic and conventional farms respectively.

### Cocoa shade strategies

In both the organic and conventional systems, the total number of shade species deployed was highest in the YCS and lowest in the OCS (S3 Table). Stem density for economic shade species was highest in the YCS and lowest in the OCS, with the MCS being intermediate. The number and stem density of shade species used for ecological purposes were also highest in YCS and lowest in OCS for the organic systems whereas on the conventional farms, they were highest in the MCS and lowest in the OCS. Shade species used for domestic purposes occurred most in OCS and least in MCS but their stem density decreased from YCS through the MSC to the OCS.

On the conventional farms, the stem density of domestic species found in the MCS was approximately twice that of the OCS and YCS though it recorded 8 shade species compared to 14 species for YCS and 9 species for OCS. A Chi-square analysis revealed that the number of species used in each tree use group was not significantly associated to either farm type or cocoa age group. However, the density of species used in each tree use group was significantly associated with both farm type (χ^2^ = 11.163, df = 2, *p* = 0.004) and cocoa age group (χ^2^ = 46.355, df = 4, *p* <0.001).

## Discussion

### Species abundance and importance value

Generally, the results show that *Musa spp*. and *Citrus spp*. were predominant on both organic and conventional farms for all the cocoa age groups but more so on organic farms. Farmers maintained these food and fruit species in larger numbers due to their economic, domestic and/or ecological benefits [7; 14]. Similarly, Tondoh *et al*. [10] also reported the dominance of fruit tree species in cocoa systems in Central Western Côte d’Ivoire and indicated that fruits trees were planted by farmers to provide shade and income.

In Ghana, Bandanaa *et al*. [8] documented 26 flora utilized for food and medicine in a study of cocoa farms in the Ashanti region, and Dawoe *et al*. [30] reported a relatively higher abundance of non-timber (fruit) trees on cocoa farms in ten districts in the Ashanti, Brong Ahafo and Western regions. The dominance of fruit trees in all these systems could be a strong indication of the deliberate transformation of the landscape by farmers from the naturally occurring pioneer species that have been traditionally grown with cocoa to species that provide food and medicinal benefits.

Apart from *Entandrophragma angolense* which is a non-pioneer light demander and a valuable commercial timber species, all the forest tree species recorded among the ten most abundant species according to the IVI on the young, mature and old organic cocoa farms were pioneer species exploited as commercial timber and for use as domestic construction materials (e.g. *Terminalia ivorensis*, *Spathodea campanulata*, *Ficus exasperata*, *Ficus sur*, *Alstonia boonei*, *Milicia regia* and *Sterculia tragacantha*) or medicines (e.g. *Newbouldia laevis*, *Holarrhena floribunda* and *Morinda lucida*).

Similarly, all the forest tree species of the young, mature and old conventional cocoa farms recorded among the ten most abundant species according to the important value index included species with medicinal values namely *Spondias mombin*, *Rauvolfia vomitoria*, *Holarrhena floribunda*, *Cola gigantea*, *Lonchocarpus sericeus*, *Voacanga Africana*, *Millettia zechiana* and *Morinda lucida* or domestic construction material and timber species namely *Ficus exasperata*, *Terminalia superba*, *Antiaris toxicaria*, *Milicia excelsa*, *Terminalia ivorensis* and *Spathodea campanulata*. The predominance of similar forest pioneer species on cocoa farms in general has been reported in Ghana [18] and in Côte d’Ivoire [10] and these authors suggested that the observed trend was a result of these species providing commercial and domestic products. Additionally, pioneer species are better able to regenerate and survive in disturbed forests or forest-like systems, such as cocoa agroforests, than other species thus contributing to their abundance on cocoa farms. Indeed, most of the species among the ten most abundant species such as *Milicia excelsa*, *Antiaris toxicaria*, *Alstonia boonei*, *Entandrophragma angolense*, *Ficus exasperata*, *Newbouldia laevis*, *Terminalia ivorensis*, *Terminalia superba* and *Spathodea campanulata* have been cited as being compatible with cocoa by both farmers and scientists in Ghana [22; 40; 41]. That notwithstanding, the vegetation in the study area has been cited to be predominantly pioneer species due to significant deforestation that has taken place over the past five decades [23; 27].

### Shade tree species richness and diversity

In general, our results show that the richest families found on the organic farms were the Sterculiaceae, Moraceae, Fabaceae and Apocynaceae and that of the conventional farms were Moraceae, Fabaceae and Apocynaceae. This observation is a reflection of farmers’ preference for tree species that provides medicine, local construction material and timber or for trees that improve soil fertility in addition to providing shade [10; 13; 16; 18]. For instance, all the species belonging to the family Apocynaceae documented in this study including *Rauvolfia vomitoria*, *Holarrhena floribunda*, *Voacanga Africana* and *Alstonia boonei* are important sources of domestic or commercial medicinal products. Tree species belonging to the families Sterculiaceae, Moraceae and Fabaceae such as *Albizia ferruginea*, *Albizia glaberrima*, *Albizia zygia*, *Amphimas pterocarpoides*, *Piptadeniastrum africanum*, *Gliricidia sepium*, *Ficus exasperata*, *Ficus sur*, *Ficus vogeliana*, *Antiaris toxicaria*, *Melicia regia* and *Melicia excelsa* improve soil nutrients through nitrogen fixation, provide quick shade, keeps soil around them cool and moist, or are important sources of both local construction material and commercial timber [13; 18; 30; 41]. Tscharntke *et al*. [16] asserts that the maintenance of high-value timber trees, such as those documented in our study, serve as a bank account for cocoa farmers and their families.

Organic cocoa farms were more diverse in terms of shade tree species than the conventional farms. The results of the present study are comparable to studies conducted elsewhere that investigated coffee [19], cocoa [7], agriculture [42] and olive groves [43], all of which found significantly higher flora diversity on organic farms compared to conventional farms. Similarly, Bandanaa *et al*. [8] reported higher species diversity on organic cocoa farms compared to conventional cocoa farms from the Ashanti region of Ghana. Several studies have demonstrated the benefits of high shade tree diversity to cocoa such as suppression of weeds and pests, host for beneficial insects, enrichment of soils and reduction of cocoa physiological stress [16; 17]; it is obvious organic farmers in our study sought to exploit these benefits through the introduction or retention of a rich list of shade trees on their farms. In general, the high species richness and diversity on organic farms compared to conventional farms show their potential for tree species conservation. The ecological zone of cocoa in major cocoa production countries significantly overlap with major biodiversity hot spots [1; 2; 16; 20]; highly diversified cocoa agroforestry in general and organic cocoa agroforestry in particular may serve as important reservoirs of biodiversity [5; 8]. Tree diversity is an important feature of both resilient farming systems [7] and climate-smart agriculture [8] and organic cocoa agroforests seems promising in this context. Certified organic cocoa enjoys premium price; organic certification as is currently being promoted would be a logical method for incentivizing agroforestry and the inclusion of diverse shade trees should be prioritised or at least be encouraged and vigorously promoted as part of the certification procedure. Furthermore, even though organic cocoa demonstrated greater potential in terms of tree species conservation, larger scale farmer adoption is required to maximise this potential. That notwithstanding, over 50% of the 1.6 million cocoa farmers in Ghana do not use agrochemicals and are classified as *de facto* organic [44; 45], thus a huge potential for organic certification already exists.

### Spatial structure and composition of organic and conventional farms

Generally, the sizes of studied cocoa farms ranged from 0.70–1.80 hectares, which converges with other studies [23; 22]. The results reveal that the stem densities of the different tree groups deployed by farmers as shade for cocoa trees were significantly associated with farm type (organic or conventional) but shade tree density and total stem density were similar on both organic and conventional farms. This suggests that for the same stem densities, organic and conventional systems make use of different densities of each tree group. For example, organic farms had twice the density of fruit trees as conventional farms which reflects an attempt by organic farmers to increase yields from co-products to supplement their cocoa yield.

The fact that cocoa tree density was significantly higher on conventional farms than the organic farms (Org. 1012 ± 40 stems ha^-1^ vs. Con. 1203 ± 40 stems ha^-1^) even though both farm types had similar total stem densities implies that the conventional farmers replaced shade and fruit trees with cocoa trees; this is a trade-off farmers often adopt [5]. The results of shade trees stem density for both organic and conventional farms diverge from those of Asare and Anders [22] possibly due to regional differences in the structure of cocoa farms [30] and the exclusion of smaller trees in their study. Shade tree and total basal areas on organic farms were consistently higher than conventional farms due to at least; (i) the retention of large old shade trees; (ii) the planting of fast-growing species; (iii) higher density of timber species; and (iv) abundant fruit trees and plants (S1 and S3 Tables).

The composition of shade species on the organic and conventional farms was dissimilar in each cocoa age group; the highest dissimilarity between organic and conventional farms was observed in the old cocoa age group. The Jaccard dissimilarity results of the present study are contrary to those reported by [8]; this is probably because the present study included older organic cocoa farms. At the early stage of organic farming, differences between organic and conventional farms in terms of species composition may be less pronounced because most organic farmers either converted from conventional cocoa systems or established them under similar forests as the conventional ones (personal observation).

### Species conservation status and ecological importance

In general, the potential of cocoa agroforests to conserve endemic, native and threatened tree species when compared to monocultures and other land use systems has been shown by several authors [3–5; 13; 16]. Our results further deepen this understanding, suggesting that shade organic cocoa may contribute more significantly to native species conservation than conventional farms due to their high shade tree diversity and the maintenance of relatively higher levels of tree species with conservation concern. For example, the number of native tree species with conservation concern which were found on only organic farms was higher compared to conventional farms (Table 1). That notwithstanding, as reported in [5], 75% of the documented trees in this study have not yet been assessed to determine their conservation status. The vegetation in the study area has been significantly transformed through deforestation into patches of secondary forests; organic cocoa agroforests may play a key role in the maintenance of native tree species and their gene pool and serve as habitats for species that tolerate some disturbances [7; 8; 16; 23; 27]. In addition to meeting the international standards for the production of organic cocoa, our studied organic cocoa agroforestry farms would also meet the criteria for Bird-Friendly, Rainforest Alliance and Fair-trade as outlined in Philpott *et al*. [46] for shade-coffee as well as chemical-residue-free UTZ. The incentives from these certification mechanisms may further motivate cocoa farmers to maintain tree biodiverse cocoa systems. However, the benefits from these other certification mechanisms must outweigh any other additional trade-off to make them desirable to cocoa farmers.

Tree pioneer species dominated young, mature and old cocoa farms in both organic and conventional systems (S2 Table). The fact that farmers retained or planted pioneers on their farms reflect the importance of these species for the provision of shade and other ecological services in cocoa agroforestry systems. Pioneers are fast growing which enables rapid establishment, canopy closure and hence provision of shade for cocoa [41; 47]. Additionally, pioneers rapidly produce large standing biomass [48], which may enhance ecological services such as carbon sequestration and nutrient recycling [4; 5; 10]. Encouraging the use of pioneer species that are already well adapted to the local ecological conditions maybe a successful approach to ensure, at low cost, the conservation of tree species, particularly pioneers with a conservation concern. Cocoa agroforestry systems are promising in this context. Furthermore, tree pioneer species in cocoa agroforestry systems represent a great opportunity to enhance plant succession as the cocoa systems mature because they can improve site conditions and attract disperser communities [4; 10; 47; 48].

### Cocoa shade strategy in studied farms

Although the number of species in each tree use group (ecological, economic and domestic) deployed by farmers was not found to be significantly associated with cocoa age group or farm type, stem density was. This suggests that the management strategy deployed by farmers of the young, mature and old cocoa farms was to manipulate the densities of a similar range of ecological, economic and domestic species to meet their own needs and that of their cocoa [5; 10; 41]. On the basis of stem density, it appears both organic and conventional farm types adopted a cocoa shade strategy that provided additional benefits but at different degrees in each cocoa age group. For example, organic farms retained relatively higher stem densities of economic shade trees than conventional farms in each cocoa age group possibly for the purpose of generating additional income.

## Conclusion

The results demonstrate that the studied organic cocoa agroforests maintain higher tree species richness, diversity and basal area compared to the conventional cocoa agroforests although both farm types had similar shade tree stem density. The most abundant forests species were predominantly pioneers with potential to provide medicinal products, timber or local construction material in addition to cocoa shade provision. The richest families on both organic and conventional farms were Moraceae, Fabaceae and Apocynaceae and fewer species (≤ 3 *spp*.) represented all the other documented families. Fruits in general and *Musa spp*. in particular dominated both organic and conventional farms but were higher in the former than the latter. The composition of shade species on the organic and conventional farms was dissimilar in each cocoa age group, with the highest dissimilarity between organic and conventional farms recorded in the old cocoa age group.

Our results indicate that organic farms retain species with conservation concern, sometimes in relatively abundant proportions, than conventional cocoa farms thus these may arguably provide some assistance in the conservation of tree species in the landscape. That notwithstanding, a large proportion of the documented trees have not yet been assessed to determine their conservation status. Farmers manipulate the densities of a range of species to meet their own needs and that of their cocoa thus they tend to use shade tree species that provides additional income. The manipulation of shade trees density which does not compromise native tree diversity as the cocoa trees age should be encouraged. The findings emphasize the potential of organic cocoa agroforests to conserve native species. However, inclusion of diverse shade trees should be required or at least encouraged in cocoa farms to realize this potential.

## Supporting information

S1 Table List of shade species and their abundance in studied cocoa systems. (CSV)

S2 Table Ecological importance of the ten most abundant shade species in organic and conventional farms across the different cocoa-age groups. (CSV)

S3 Table The number and stem density of shade species used for domestic, ecological and economic purposes. (DOCX)
