# Supplementary material for: Tree diversity and its ecological importance value in organic and conventional cocoa agroforests in Ghana
Source: PLoS One. 2019 Jan 11;14(1):e0210557. doi: 10.1371/journal.pone.0210557 (PMC6329512; doi:10.1371/journal.pone.0210557)
Supplement: S3 Table — (DOCX) [file pone.0210557.s003.docx]

**S3 Table: The number and stem density of shade species used for domestic, ecological and economic purposes**

|  |  | **Tree use group** | | |
| --- | --- | --- | --- | --- |
| **Cocoa age group** | **Farm type** | **Domestic** | **Ecological** | **Economic** |
| **Number of shade species** | | | | |
| YCS | Organic | 10 | 13 | 18 |
|  | Conventional | 14 | 10 | 17 |
| MCS | Organic | 9 | 11 | 18 |
|  | Conventional | 8 | 12 | 15 |
| OCS | Organic | 13 | 5 | 17 |
|  | Conventional | 9 | 5 | 13 |
| **Shade species stem density** | | | | |
| YCS | Organic | 44.57 | 30.86 | 443.43 |
|  | Conventional | 35.43 | 18.29 | 388.57 |
| MCS | Organic | 35.43 | 23.00 | 284.57 |
|  | Conventional | 66.29 | 26.29 | 122.29 |
| OCS | Organic | 33.14 | 11.43 | 163.43 |
|  | Conventional | 17.14 | 17.14 | 96.00 |
